# Silicon quantum dots inlaid micron graphite anode for fast chargeable and high energy density Li-ion batteries

**DOI:** 10.3389/fchem.2022.1091268

**Published:** 2022-12-06

**Authors:** Huanxin Li, Mark A. Buckingham

**Affiliations:** ^1^ Department of Engineering, University of Cambridge, Cambridge, United Kingdom; ^2^ Department of Materials, The University of Manchester, Manchester, United Kingdom

**Keywords:** silicon quantum dots, micron graphite, fast chargeable, high capacity, Li-ion batteries

## Abstract

The pursuit of rapid charging and high energy density in commercial lithium-ion batteries (LIBs) has been one of the priorities in battery research. Silicon-Carbon (Si-C), a possible substitute for graphite as an anode electrode material, is one prospect to achieving this goal. There is a debate as to whether nanoscale or the micron-scale silicon is more favourable as anode materials for LIBs. Micron-scale silicon exhibits relatively higher initial coulomb efficiency (CE) compared with nanoscale silicon, while its cycle stability is poorer. However, minimizing silicon normally benefits the cycle stability, but introduces serious side reactions, due to the large active surface for nanoscale silicon. Here, we propose silicon quantum dots (Si QDs) inlaid in micron graphite (SiQDs-in-MG) as an anode for high energy density and fast charging LIBs. The Si QDs almost eliminate the volume change typically observed in Si during long-term cycling, while the graphite blocks solvent entering the channels and contacting the SiQDs, promoting the generation of a stable solid electrolyte interphase, which is not in direct contact with the Si. SiQDs-in-MG addresses the main issues for Si-based anodes and is expected to achieve high energy density when in combination with a Lithium-Nickel-Manganese-Cobalt-Oxide (NMC) cathode in pouch cells.

## Introduction

Increasing global populations, coupled to increasing industrialisation of developing nations is leading to a significant increase in global energy demands. As non-renewable energy sources such as coal, oil, and gas lead to increasing greenhouse gas emissions, there is a current drive towards the generation of renewably generated energy. However, the currently targeted renewable energy conversion systems such as wind and solar are intermittent. To combat this, sustainable and reliable energy storage devices are required to store renewably generated energy, which can be used during periods without the ability to utilise wind or solar. Electrochemical energy storage devices such as batteries and capacitors realize storage of energy to high capacities and allow continuous output when required.

Lithium-ion batteries (LIBs) have dominated the battery market since the first successful commercialization in 1991 (Kamat, 2019). Currently, most mobile phones, computers and electric vehicles use LIBs as power sources. Grid-scale lithium-ion batteries have also been implemented, most notably in the Hornsdale power reserve in South Australia. Electric vehicles (EVs) also utilise lithium-ion batteries and are increasing globally. This increase in EVs is driven by policies such as the United Kingdom banning the sale of new petrol and diesel cars by 2030. Although LIBs are already critical towards achieving renewable energy targets, there are still challenges. Technical challenges such as charging rate, endurance time/mileage and safety still need to be surmounted to satisfy expectations and demands of users (Duan et al., 2020). With the increasing popularity of EVs, these challenges are becoming increasingly prominent. For instance, the time-consuming charging process sacrifices the convenience of EVs compared to traditional fuel vehicles. Rapid charging of LIBs, aiming at shortening the recharge times to ∼10–15 min is crucial for EVs to become competitive with the fuel vehicles. The US Department of Energy announced support for projects targeted at increasing charging station power to 400 kW, which could shorten the charging time of a 60 kWh EV to ∼10 min (Yang et al., 2018). The United Kingdom government recently released a protocol to boost the rapid charge-points (150–350 KW capable) with the capability to charge 3 times faster than most of the current chargepoints, ensuring the delivery of around 120–145 miles within just 15 min for a typical EV, with around 6,000 high powered charge-points across England’s motorways and major roads expected to be established by 2035 (Monika et al., 2022). The Rapid Charging Fund was announced in the March 2020 Budget as part of a £500 million commitment for EV charging infrastructure. The purpose of this programme is to ensure a rapid-charging network ready to meet the expected long-term consumer demand for EVs. In addition, automobile companies in Europe plan to deploy 350 kW fast charging stations across Europe (Sarah and Ryan, 2022).

As rapid charge-points become widely implemented, the LIBs in EVs will also have to improve in order to become compatible. It is therefore of urgent interest to develop LIB technologies for high safety, fast charging rates, and long endurance in performance. However, the safety performance, charging rate and endurance time of LIBs are not independent variables and alterations in one often effect one of the other parameters. To obtain a balance, it is necessary to comprehensively consider the electrode design and optimize technical parameters during battery assembly. To date, the development of LIBs with excellent safety performance, fast charging rates, and long endurance times mainly faces the following challenges: Firstly, the rapid-charging network could shorten the charging time but intensifies the polarization of the battery and generates significant quantities of thermal energy, resulting in capacity loss and increased risk to safety through overheating. Secondly, to achieve a long endurance time for LIBs, the energy density needs to be increased, this will also sacrifice the charging rate and cause certain safety hazards, such as high heating, causing fires. Thirdly, in order to ensure high safety standards, the energy density and charging rate of LIBs are restricted in practical applications (for the reasons mentioned above). To address these issues simultaneously, the design of novel electrode materials with good compatibility in LIBs, as well as the optimization of the technical parameters of the whole battery (e.g., areal loading, N/P ratio, capacity ratio between the negative and positive electrodes) are urgently required.

## Fast chargeable anode

There are many factors that effect the charging rate of LIBs. Typically, the following four steps will be proceed during the transferring of Li^+^ ion from the electrolyte to the electrode material: 1) The solvated Li^+^ ions migrate from the electrolyte to the surface of the electrode material; 2) The de-solvation process is completed at the solid electrolyte interphase (SEI) normally coated on the surface of the electrode; 3) The de-solvated ions enter the matrix of electrode material through the SEI; 4) Lithium ions migrate within crystal channels in the electrode material. Different types of LIBs may have different rate-determining steps under different conditions (Cai et al., 2020). Current research into migration mechanisms of LIBs has determined that in LIBs, the mechanism for the pathway of lithium ions are notably different between the cathode and anode electrode materials, resulting in a higher migration rate (around two orders of magnitude) for lithium ions moving in the cathode electrode than that in anode electrode (Xie et al., 2019). Therefore, the anode often dominates the charging rates of LIBs. It is particularly important to design and prepare anode electrode materials that allow rapid Li^+^ ion migration for the construction of fast charging LIBs (Son et al., 2017; Yao et al., 2019; Zhu et al., 2019). Although some studies have shown that layered graphite can achieve a degree of rapid charging, it has thus far eluded researchers to completely eliminate the negative impact of rapid charging in LIBs, such as Li dendrite formation, graphite exfoliation, and increasing battery resistance. Lithium dendrites are formed by the rapid deposition of Lithium at the electrode surface above the rate of intercalation into the storage electrode, forming dendritic lithium. This process is more prominent at greater charging rates and drastically shortens the service life of the battery and substantially affects its safety performance due to the higher overpotential. This is also an important factor that restricts rapid charging of LIBs.

**FIGURE 1:**
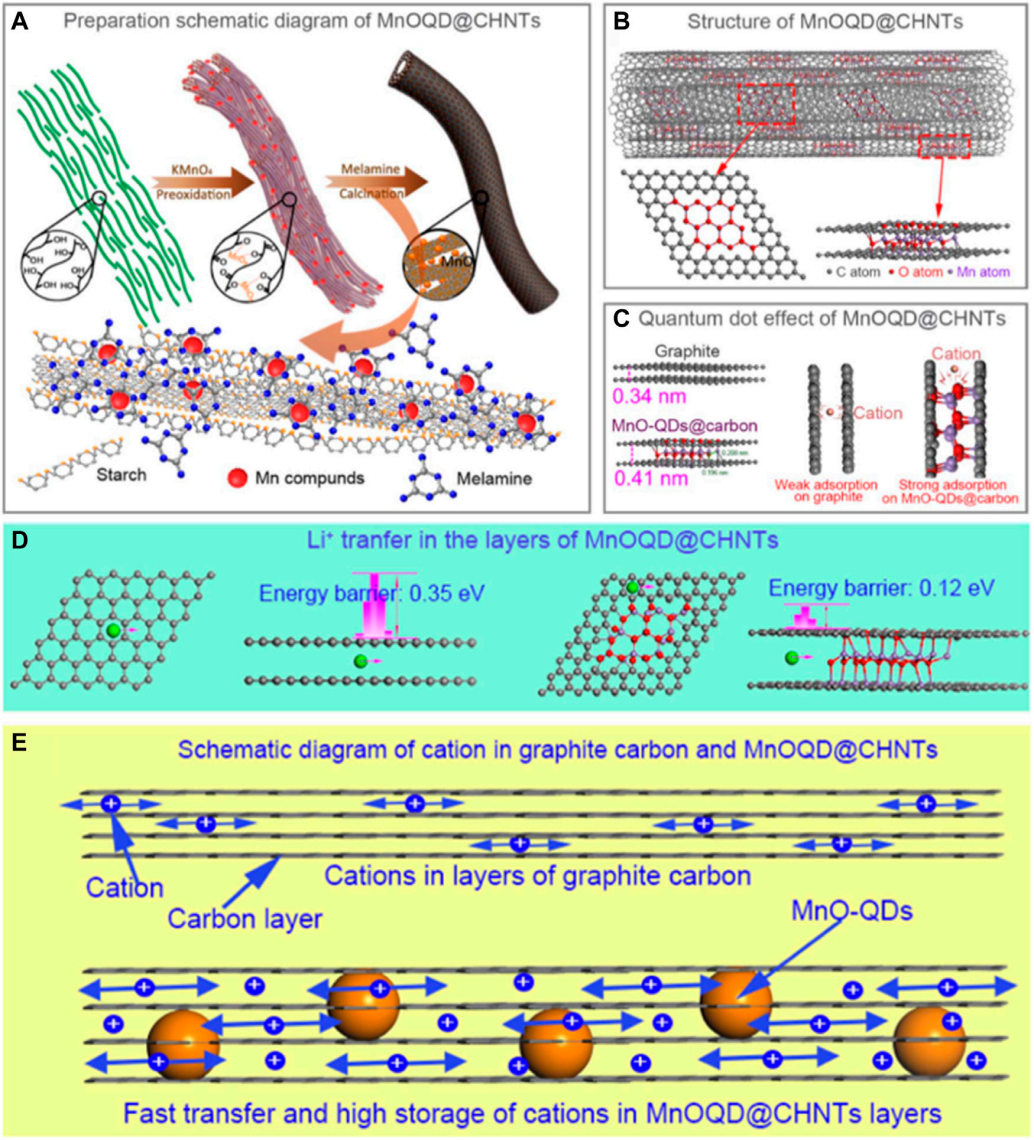
**(A)** Preparation schematic diagram, **(B)** structure and **(C)** quantum dot effect of MnOQD@CHNTs; **(D)** Energy barriers of Li^+^ ion migrating in the layers of graphene and MnOQD@CHNTs; **(E)** Schematic diagram represents that cations can be strongly adsorbed and rapidly transport in MnOQD@CHNTs. Copyright from Elsevier (Li et al., 2019).

Through electrode structure design at the nanoscale, the layer spacing of graphite could be modulated (widened), a wider inter-layer spacing of the graphene sheets in graphite can notably reduce the energy barrier towards Li^+^ intercalation, decreasing the resistance of Li^+^ ion insertion and migration. In order to control the layer spacing of graphite nanomaterials, precise nanoscale structural design should be carried out. Our group has previously conducted preliminary work for inlaying quantum dots (QDs) to control the layer spacing of graphitic nanomaterials, for example using MnO (Feng et al., 2018; Li et al., 2019), Fe_3_C-TiN (Li et al., 2021), Ni_2_P (Yan et al., 2021a), FeS (Yan et al., 2021b) and other quantum dots inlaid in graphite have been prepared. The layer spacing of these graphite nano-materials were expanded, from ∼0.34 nm for the original graphite to ∼0.41 nm (depending on the nature of the QD) after the modification with QDs and the physical and chemical properties (electronic conductivity, ion mobility, *etc.*) have been largely altered, ascribed to the heterojunction formation between graphite and QDs. The MnO QDs-modified system delivers a capacity up to 400 mAh g^−1^ under an ultra-high current density of 50 A g^−1^ (rapid charging in 28 s) when used as anode electrodes for LIBs, which is the state-of-the-art anode material for LIBs (Li et al., 2019). The above work shows that QD-modified graphite nano-composites are a promising potential route towards rapidly chargeable LIBs (Figure 1).

Based on this concept, high-capacity QD-modified graphite is expected to achieve high lithium storage capacity and faster charging ability. Here, we propose a SiQDs-in-MG (micron-scale graphite) as the anode of rapid chargeable and high-capacity LIB. Si QDs would undoubtedly provide extra capacity and expand the layer spacing when inlayed into graphite, which will again notably reduce the resistance for Li^+^ ion insertion/migration and thereby minimize polarization. Meanwhile, the Si QDs would marginally increase the deposition voltage of Li^+^ ions (∼0.3 V vs*.* Li^+^/Li), eliminating the risk of Li dendrite formation on the electrode surface. Therefore, the SiQDs-in-MG, with a designed structure and unique property (enlarged layer spacing, minimized Si diameter), has the potential to be a promising anode for rapidly chargeable LIBs.

## Discussion

Si-C-based anodes are a promising substitute for the graphite extensively used in LIBs, and are promising in the applications of rapid charging EVs. The theoretical capacity of Si is 10-fold higher than natural graphite, which ensures a high energy density for LIBs that utilise Si-C anodes (McBrayer et al., 2021). Sharma and Seefurther are the first to prove the feasibility of lithium silicon alloy as anode for LIBs in high-temperature molten salt electrolyte (Sharma and Seefurth, 1967). In the early 1980s, Wen and Huggins identified various compositions of lithium silicon alloy by coulometric titration, and determined that the maximum theoretical specific capacity of silicon is 4200 mAh g^−1^ at high temperatures (415°C) (Wen and Huggins, 1981). In 1995, Dahn and co-workers synthesized a silicon carbon composite electrode containing 11% atomic silicon, with a specific capacity of 600 mAh g^−1^ (Wilson et al., 1995). It was not until 1999, Chen et al. prepared a composite of silicon nanoparticles and carbon black with a specific capacity of 1700 mAh g^−1^ (Li et al., 1999). Then, Cui et al. developed silicon nanowires grown directly from the metal collector as anode in 2008 (Chan et al., 2008), and a company named Amprius Inc. was founded to commercialize the silicon-nanowire-based anode. After that, various nanostructured silicon based anode materials (silicon nanotube, micro silicon) have been extensively investigated and reported (Magasinski et al., 2010; Li et al., 2016; Xing et al., 2018; Zhang et al., 2021). As mentioned above, the reduction voltage of Si vs*.* Li^+^/Li is slightly less cathodic than that of graphite, alleviating the formation of Li dendrites on electrode surfaces, even under rapid charging polarization. In addition, silicon is a highly abundant element within the Earth’s crust, whilst natural graphite is far less abundant, being considered a critical raw material (Jędrzej et al., 2021).

Despite the advantages of Si-C anodes, there are some issues that have hindered the commercialization of Si-C. Such as the large volume change (∼300%) of Si during the charging-discharging processes, which provides a high risk of inflating for pouch cells of LIBs. Side reactions between the Si surface and electrolyte produce undesirable gaseous evolution, and the pulverization of Si during the volume changing process will refresh the surface of Si and facilitates further side reactions. The semiconducting nature of Si limits the electronic conductivity, reducing the effectiveness of the battery electrodes. It is still unknown if the nanoscale or the micron-scale silicon is more advantageous to the practical application as an anode material for LIBs. Micro-scale silicon exhibits relatively higher initial coulomb efficiency (CE) compared to nano-scale silicon, due to the lower surface area, while the micro-scale silicon has poorer cycling stability due to the large volume changing. However, reducing the size of silicon normally benefits the cycle stability, but introduces detrimental side reactions, such as HF generation, owing to the large active surface for nano-scale silicon (Chaudhry et al., 2018).

SiQDs-in-MG is expected to address the disadvantages ascribed to Si-C discussed above. This is due to the Si QDs alleviating the volume change during cycling, while the graphite reduces the electrode surface and promotes the generation of a stable solid electrolyte interphase, not involving the Si. Since QDs maintain the favourable intrinsic properties of nano materials, but over a small area, they maximize advantages such as large electrochemically active surface area, short diffusion pathways for ions, and minimised volume changing. The micro-graphitic carbon protects the Si QD surface, whilst also eliminating side reactions in most circumstances.

## Concept of SiQDs-in-MG

**FIGURE 2:**
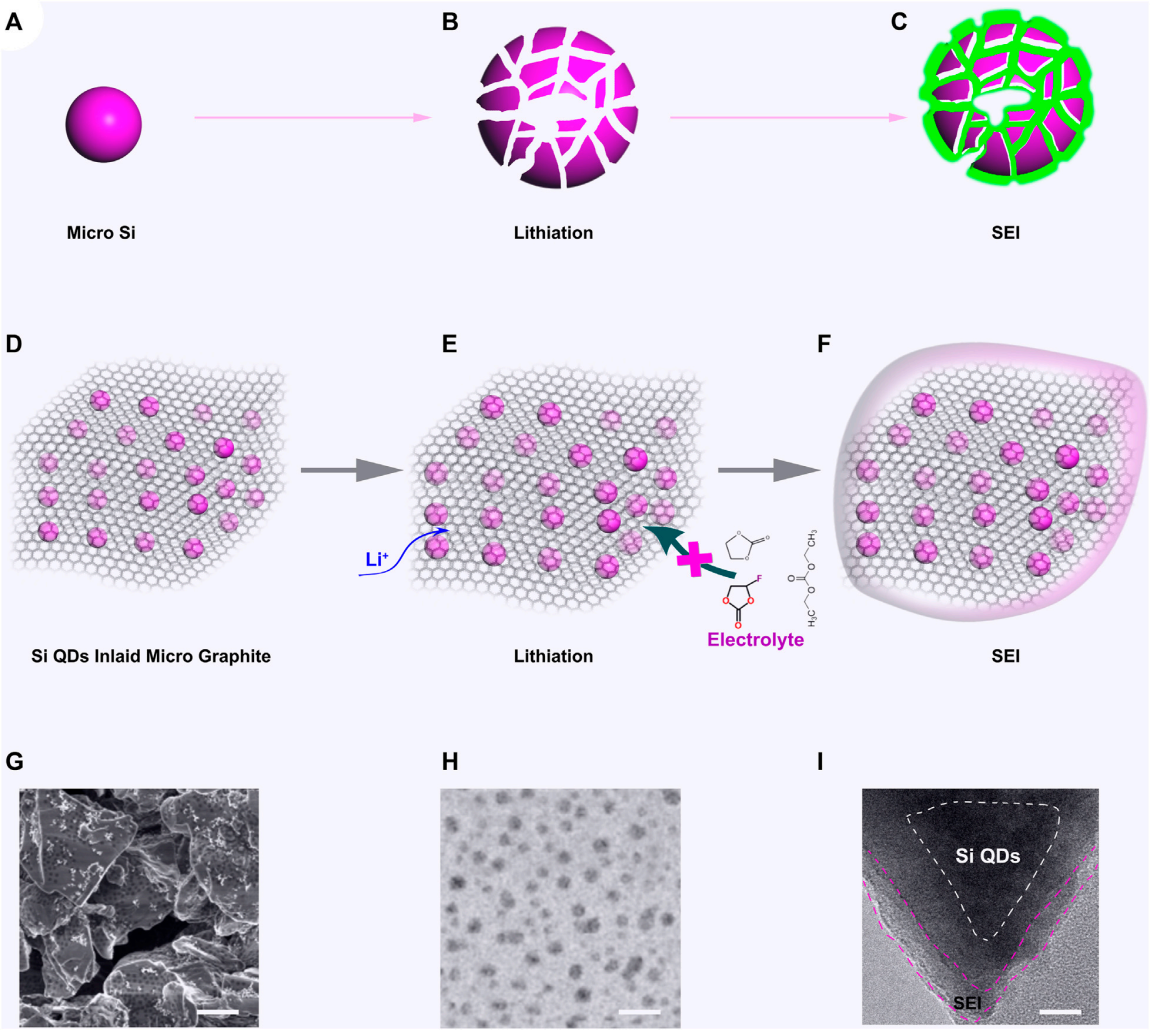
**(A)** The micron Si; **(B)** Micron Si after lithiation; **(C)** SEI formation on lithiated Si; **(D)** SiQDs-in-MG; **(E)** SiQDs-in-MG after lithiation; **(F)** SEI formation on SiQDs-in-MG; **(G)** SEM image of SiQDs-in-MG, scale bar 5μm; **(H)** TEM image of SiQDs-in-MG, scale bar 10nm; **(I)** TEM image of SiQDs-in-MG after SEI formation, scale bar 2 μm.

A schematic for the lithiation processes for micron Si and SiQDs-in-MG in LIBs are shown in Figure 2. As shown, the micron Si (Figures 1A–C) is notably expanded during lithiation, resulting in a breakup of material (Figure 1B). This results in the SEI continuously increasing as the material breaks apart, forming an increased Si surface area (Figure 1C). This process results in poor stability, low coulomb efficiency (CE), and gaseous (H_2_, CO_2_, CH_4_, C_2_H_4_, C_2_H_6_) evolution issues for LIBs. In SiQD-in-MG (Figure 1D), the nano channels in micron graphite are ∼0.4 nm (slightly expanded compared to natural graphite due to the modification with Si QDs). The modified graphite acts as a selective nano channel between the Si QDs and LIB electrolytes as the de-solvated Li^+^ ions will pass through the micron graphite via the nano channels, while the solvent molecules are blocked due to their larger size and therefore cannot reach the Si surface, preventing any undesirable side reactions. This process both protect the Si QDs also preventing the volume changing typically observed with Si (Figure 1E).

In the SiQD-in-MG, due to the expanded graphite layer from incorporating Si QDs between the layers, high electrical conductivity between Si QDs and graphite layers are achieved, whilst the pulverisation of Si from expansion (Figures 2A,B), and unfavourable Li dendrite formation are expected to be eliminated. The micron graphite reduces the surface area of Si QDs, and a stable SEI could be formed on the surface of SiQD-in-MG, thereby improving the CE over extended timescales (Figure 1F). The preparation of SiQD-in-MG is feasible by several methods, including wet chemical approaches, magnetron sputtering and high-temperature thermal treatments. Figures 1G,H shows SiQD-in-MG synthesized by pyrolysis. Both scanning electron microscopy and transmission electron microscopy imaging indicate that Si QDs were uniformly dispersed in the MG at both the microscale and nanoscale. After lithiation, the SEI formed on the surface of the MG, while the Si QDs were well-protected, without an SEI being formed between Si and electrolyte.

## Fabrication of full pouch cells

Although Si-C anode electrodes have attracted significant attention, these materials are still in the early stages of development at the laboratory stage and have not been widely applied in the commercial LIBs. Despite achievements for Si-C anodes in LIB (the reported specific capacity is in the range of 400-600 mAh g^1^), there remain difficulties in practical pouch cell preparation. The poor adhesion of Si with binders as electrode materials makes the capacity decay rapidly. The contact of silicon with electrolyte will promote the hydrolysis of [PF_6_]^-^ and continuously produce corrosive and highly toxic hydrofluoric acid (HF), which normally results in electrode corrosion and gaseous evolution within the cell (Li et al., 2021). The graphite in SiQD-in-MG anodes protect the Si QDs and avoid a direct silicon and electrolyte boundary by forming a stable SEI on the protecting graphite, which not only reduce side reactions, but also eliminate the gaseous evolution, and alleviate the poor adhesion between Si, binder, and current collectors, improving long-term stability.

The combination of Si-C anode and high nickel (up to 80%) Li-Ni-Mn-Co-oxide (NMC) cathode (e.g., NMC811) to achieve high energy densities for next generation LIBs has already been recognized as a promising technology (Chaudhry et al., 2018; Tan et al., 2021). For this, the SiQD-in-MG anode and the NMC ternary cathode electrodes could be an excellent combination for assembled pouch cells, as to maximise the advantages for both anode and cathode materials. The ideal negative/positive capacity (N/P) ratio is ∼1.4, given a capacity for SiQD-in-MG of ∼1200 mAh g^1^. With further optimization of the electrodes, electrolytes and interfaces, it is expected to develop LIBs with exellent safety performance, fast charging rates, and long endurance time and cycle life (Self et al., 2015; Pang et al., 2019; Pang et al., 2020; Xue et al., 2020).

## Perspective

According to previous research (Li et al., 2019), the suitable diameter for silicon QDs in graphite is in a range of (1–10 nm). There is a wide array of possible QDs that can be incorporated into MG, such as elemental, metal oxides, chalcogenides, or others (nitrides, phosphides) and even more complex materials such as high-entropy materials. This tunability provides substantial flexibly and control for the final fabricated material (Yang et al., 2022). Given that Si QDs could be synthesized to a uniform dispersion within the graphite, it is expected that a novel anode for LIBs provided by SiQD-in-MC could be a material that effectively addresses the issues discussed above.

In summary, SiQD-in-MG have several advantages. Firstly, Si QDs can effectively regulate the graphite carbon layer spacing to reduce the ion insertion/migration energy barrier, as to obtain a Si-C based anode electrode material that allows rapid migration of lithium ions. Secondly, the host graphite can effectively protect the Si QDs and avoid the irreversible side reaction between the electrolyte and the active silicon; Finally, the finely tuned graphite layer spacing with QD incorporation allows lithium ions to selectively and rapidly permeate the graphite and react with the Si QDs, whilst being impermeable to the supporting electrolyte to form a stable SEI. With promising capacity, safety, and the potential for rapid charging. Due to the connection between Si QDs and micron graphite, the short ion transfer path in QDs and the slightly higher reduction voltage than graphite, which mitigates against the formation of Li dendrites. Based on precise design and preparation of devices, the SiQD-in-MG LiBs are expected to achieve rapid charging, high CE, long cycle lifetimes, and good safety performance. Future work in this area should be focused on reducing cost and scaling up production.

## Data Availability

The original contributions presented in the study are included in the article/supplementary material, further inquiries can be directed to the corresponding author.
